# Insect Peroxiredoxins: A Comprehensive Review of Their Classification, Distribution, Structural Features, Expression Profiles and Physiological Functions

**DOI:** 10.3390/insects16070678

**Published:** 2025-06-28

**Authors:** Li Yang, Shaohua Lu, Yujie Lu, Mingshun Chen, Sufen Cui

**Affiliations:** 1 School of Food and Strategic Reserves, Henan University of Technology, Zhengzhou 450001, China; yangli139039@163.com (L.Y.); luyujie1971@163.com (Y.L.); 2 School of Grain Science and Technology, Jiangsu University of Science and Technology, Zhenjiang 212100, China; sufen18@just.edu.cn; 3 Department of Entomology, Kansas State University, Manhattan, KS 66506, USA; mchen@ksu.edu

**Keywords:** peroxiredoxins, cysteine, antioxidant defense, physiological functions, pest control

## Abstract

With the development of technology, an increasing amount of literature regarding the expression profiles and physiological functions of Prxs has been published. Despite this growing interest, there is currently no systematic review of expression profiles in different insects. Here, we performed a systematic review of the available literature on the location and expression of Prxs in different tissues, developmental stages and sexes in insects. Recent studies on the structure, expression profiles and functional characterization of Prxs provide valuable insights into the molecular mechanisms and functional pathways of this important enzyme family. In insects, Prxs are crucial for antioxidant defense, development, stress adaptation, cell apoptosis, immune response and insecticide resistance. This systematic review provides an overview of the various functions of Prxs as reported in the literature and highlights that many environmental stresses induce changes in Prxs expression levels. Furthermore, we present perspectives on future research directions regarding insect Prxs and discuss their potential applications in pest control.

## Simple Summary

Peroxiredoxins (Prxs), initially identified in *Saccharomyces cerevisiae*, are a superfamily of cysteine-dependent peroxidases that play a crucial role in the defense against oxidative stress in various organisms, including insects. Prxs protect cells from oxidative damage by neutralizing reactive oxygen species (ROS), which are byproducts of normal cellular metabolism. Prxs have been found to be widely expressed in various developmental stages and tissues of insects, suggesting that Prxs perform different physiological functions. This review summarizes the nomenclature, structure, classification, expression profiles and physiological functions of Prxs in insects. This comprehensive understanding of Prxs in insects not only aids in the development of new pest management strategies, but also contributes to a broader understanding of insect biology and ecology.

## 1. Introduction

Reactive oxygen species (ROS), including superoxide anion (O_2_^·−^), hydrogen peroxide (H_2_O_2_), the hydroxyl radical (·OH) and singlet oxygen (^1^O_2_), are byproducts of normal metabolic activities and are also produced under stress conditions in various organisms. ROS play essential roles in cell signaling and the induction of host defense genes [1,2]. Living organisms face a variety of biotic and abiotic stresses, such as environmental changes and infectious pathogens. Through long-term evolution, organisms have developed a range of mechanisms to overcome adverse conditions. When these adverse factors are detected, ROS are rapidly generated in organelles, and the signal is subsequently transmitted to help organisms adapt to the changing environment. ROS are then efficiently removed to prevent oxidative damage. As the most diverse group in the animal kingdom, insects inhabit highly complex environments. To mitigate ROS-induced cell damage, insects have developed an antioxidant enzyme system, which includes superoxide dismutases (SODs), catalases (CATs) and peroxidases (PODs) to combat oxidative stress and mitigate its detrimental effects [3].

Peroxiredoxins (Prxs), also known as thioredoxin peroxidases (TPxs), constitute a large family of thiol-specific antioxidant enzymes found in various prokaryotic and eukaryotic organisms including insects [4]. Prxs function as antioxidants through their peroxidase activity, catalyzing the reduction of H_2_O_2_, peroxynitrite, and a broad range of organic hydroperoxides (ROOHs) to alcohol (ROH) and water (H_2_O). Thus, Prxs play a critical role in protecting insects from both biotic and abiotic stresses, such as microbial infections, extreme temperatures, insecticide exposure, abnormal levels of H_2_O_2_ and other oxidative stresses induced by intracellular ROS [5].

Over the past few decades, many scientists around the world have conducted extensive studies on Prxs, most of which have focused on mammals and plants. In mammals, Prxs have been shown to modulate signaling cascades involved in cell differentiation, proliferation and immune responses, in addition to providing direct antioxidant protection [6]. Since insects inhabit diverse environments and experience various stresses during their life cycles, research on insect Prxs can provide unique insights into their functions and molecular mechanisms. This review synthesized current knowledge on insect Prxs, briefly describing their nomenclature, structural features and classification while highlighting divergent functional mechanisms. We evaluate expression profiles across developmental stages and tissues and discuss physiological roles in stress adaptation, immunity, and longevity. By integrating comparative analyses with structure and function, we propose their potential as targets for innovative pest management strategies.

## 2. Discovery and Nomenclature of Prxs

Prx was initially identified in *Saccharomyces cerevisiae* [7]. In addition to its protective activity, yeast Prx protects cells from damage caused by the thiol-dependent mixed-function oxidation (MFO) systems. Initially, yeast Prx was believed to primarily remove reactive sulfur species, such as RS^·^, RSSR^·−^ and RSOOH rather than ROS. As a result, it was named a protector protein (PRP) or thiol-specific antioxidant (TSA) [7,8]. However, these thiol-dependent peroxidases were later found in all organisms, with multiple isoenzymes existing in individual species. Unlike other enzymes known at the time to remove ROS, purified TSA lacked redox cofactors. In 1990, a high-sequence homology between TSA and alkyl hydroperoxide reductase (AhpC), identified in *Salmonella typhimurium*, was discovered, suggesting that TSA might also function as a peroxidase through a mechanism similar to AhpC [9]. Subsequently, TSA was found to reduce peroxides using thioredoxin (Trx) as the immediate hydrogen donor. As a result, the name TSA was changed to TPxs [10]. Later, they were renamed Prxs after it was discovered that certain members, such as the 1-Cys Prxs, did not rely on Trx as an electron donor [10].

## 3. Structure and Classification of Prxs

**Figure 1:**
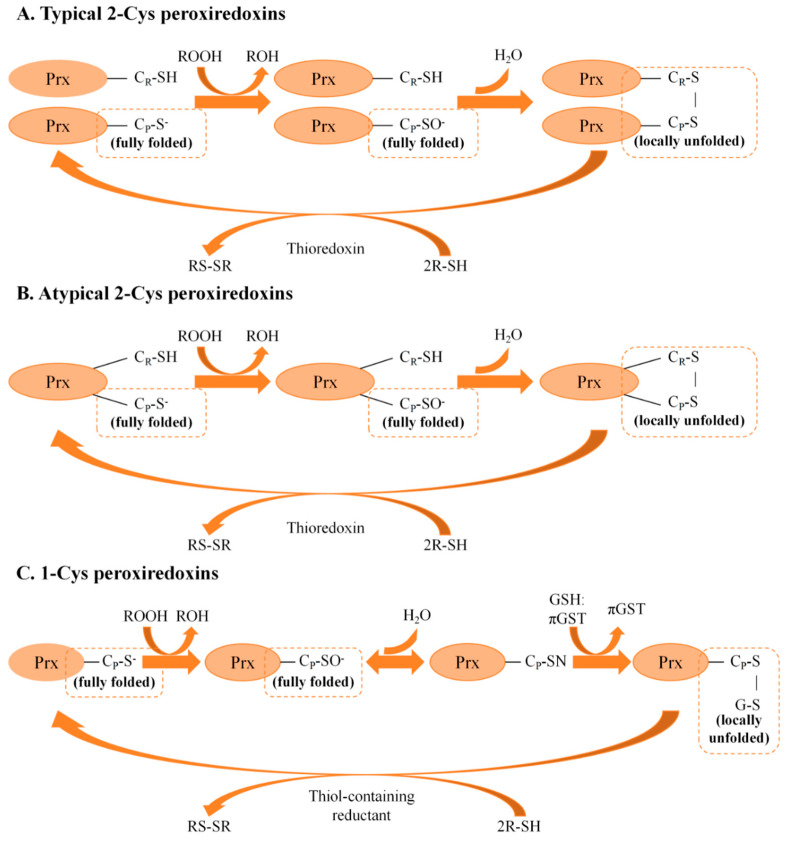
The reaction mechanisms of the three types of Prxs. Peroxide reduction by Prxs involves three main chemical steps of peroxidation, resolution and recycling. Two distinct protein conformations are involved in the cycle: fully folded (active-site intact) and locally unfolded (disulfide between the C_P_ and the C_R_). (**A**) The reaction mechanism of typical 2-Cys Prxs. The reactions catalyzed by typical 2-Cys Prxs require the formation of a dimer. In the case of dimeric 2-Cys Prxs, the C_P_ and C_R_ originate from different subunits and condense to form an intersubunit disulfide bond. (**B**) The reaction mechanism of atypical 2-Cys Prxs. For atypical 2-Cys Prxs, the oxidized C_p_ interacts with the C_R_ residue in the same subunit. (**C**) The reaction mechanism of 1-Cys Prxs. Sulfenic acid is formed by the C_P_ residue in 1-Cys Prxs, which is supplied directly by transferring an electron to the thiol in interaction with ascorbate [15].

Cysteines (Cys) are highly conserved in proteins and are predominantly located in functionally or structurally critical regions, where they serve as stabilization, catalytic, metal-binding and/or redox-regulatory entities [11]. Based on their distinct enzymatic mechanism and the specific cysteine set engaged in their catalytic cycle, Prxs are classified into two subfamilies: 1-Cys Prxs and 2-Cys Prxs [12]. The primary distinction between 1-Cys and 2-Cys Prxs is the number of cysteine residues involved in the catalytic cycle. 1-Cys Prxs contain a single conserved peroxidatic cysteine (C_P_) residue at the N-terminus, which is highly reactive to H_2_O_2_. This cysteine performs the initial nucleophilic attack on H_2_O_2_, resulting in the formation of cysteine sulfenic acid (C_P_-SOH) and the release of water. In contrast, 2-Cys Prxs possess both the conserved C_P_ residue at the N-terminus and a conserved resolving cysteine (C_R_) residue at the C-terminus [13]. Based on the mode of disulfide bond formation, 2-Cys Prxs are further classified into typical and atypical 2-Cys Prxs [14]. In addition to the C_P_ at the N-terminus and the C_R_ at the C-terminus, atypical 2-Cys Prxs possess cysteine residues at other sites, often forming intramolecular disulfide bonds. In typical 2-Cys Prxs, however, the C_P_ of one subunit forms an intermolecular disulfide bond with the C_R_ of the other. The enzymatic mechanisms catalyzed by typical 2-Cys, atypical 2-Cys and 1-Cys Prxs are shown in Figure 1.

The catalytic cycle for the reduction of hydroperoxides by Prxs involves three main chemical steps: peroxidation, resolution and recycling [16]. The catalytic cycle begins with the peroxide substrate binding to the fully folded active site. In this conformation, the enzyme has a fully formed peroxide-binding active site and the C_P_-SH is activated and ready to react with the substrate. In the peroxidation step, the hydroperoxide substrate is reduced, resulting in the oxidation of the reactive Cys moiety to C_P_-SOH. In the resolution step, C_R_-SH attacks C_P_-SOH to release a water molecule and form a disulfide. For this attack to occur, the C_P_-SOH group must move out of the protected fully folded active site pocket. This involves a conformational change, resulting in a locally unfolded conformation (Figure 1). In the fully folded conformation, the conserved C_P_ residues is located in the first turn of the α2 helix, and C_R_ is positioned in a β-strand at the C-terminus extension of the adjacent subunit. To form a disulfide bond, both motifs have to undergo a local unfolding in order for the cysteines to approach. The fully folded and locally unfolded conformations are expected to be in dynamic equilibrium until the formation of the disulfide bond in the resolution step locks the protein in a locally unfolded state and prevents the fully folded state from reforming. The recycling step occurs when the disulfide bond is broken down by another protein or a small molecule thiol, which regenerates the free thiols C_P_-SH and C_R_-SH. Although the initial peroxidation step appears to be similar for all Prxs types, the subsequent resolution and recycling steps differ between 2-Cys Prxs and 1-Cys Prxs (Figure 1). The catalytic processes also vary between typical and atypical 2-Cys Prxs [17]. In typical 2-Cys Prxs, peroxides oxidize C_P_-SH to form C_P_-SOH, which then reacts with the C_R_ residue in the other subunit, forming an intermolecular disulfide bond. This disulfide is subsequently reduced by an electron donor, completing the catalytic cycle [17]. For atypical 2-Cys Prxs, the C_P_-SOH reacts with the C_R_ residue in the same subunit, resulting in the formation of an intramolecular disulfide bond. 1-Cys Prxs, also classified as Prx6, represent a unique category of Prxs, distinguished by the absence of the C_R_ [18,19]. Beyond their well-characterized peroxidase functions in H_2_O_2_ scavenging, insect Prxs exhibit developmental stage- and tissue-specific expression patterns [20] and play crucial roles in redox signaling and chaperone activity during stress. Notably, the hyperoxidation of C_P_ to sulfinic (-SO_2_H) or sulfonic (-SO_3_H) acids, a key regulatory mechanism in mammals [13], remains understudied in insects. In 1-Cys Prxs, the sulfenic acid formed on the C_P_ residue is reduced by a heterologous thiol-containing reductant [21]. In this process, the C_P_ residue in the peptide chain can be oxidized to C_P_-SOH, which drives the reaction and reduces the levels of ROS [22]. The reformation of the peroxide-binding active site after disulfide reduction in the recycling step requires local refolding events. For this reduction to occur, other thiol-containing molecules are required to act as hydrogen donors. In the case of 1-Cys Prxs, C_P_-SOH may then react directly with other thiols, such as GSH, leading to the formation of a disulfide bond [23].

As more and more members of the Prx family were discovered and more detailed studies were conducted, various classification systems were proposed. In 2010, some scholars proposed the concept of “global evolutionary taxonomy” [19,24]. Using the Deacon Active Site Profiler tool, Prxs were classified into six groups: AhpC-Prx1, BCP-PrxQ, Prx5, Prx6, TPx and AhpE [19]. The AhpC-Prx1 subfamily is essentially synonymous with the “typical 2-Cys Prxs” [25]. This subfamily also includes Prx I–IV in mammalian cells, Bas1 in plants, AhpC in bacteria and TSA in yeast. Members of the AhpC-Prx1 subfamily play a key role in cellular signal transduction [26], and Prx1 may modulate peroxide signaling by consuming the reducing power accumulated in the cell in the form of thioredoxins, thus competing with other Trx-dependent signaling proteins. Some of AhpC-Prx1 subfamily members are regulated by phosphorylation [27]. A threonine residue at the α3 helix of the mammalian Prx1-typ peroxiredoxins can be phosphorylated, which leads to the attenuation of peroxidase activity, probably by destabilization of the decamers [6]. In mammalian cells, PrxV and PrxVI belong to the Prx5 and Prx6 subfamilies, respectively, while the TPx and BCP-PrxQ subfamilies are not expressed in mammalian cells. The oligomeric states of these enzymes are also very diverse: monomers, dimers, decamers and other quaternary species. Decameric peroxiredoxins show higher catalytic efficiency than dimers. Prxs exhibit different catalytic patterns according to their subfamilies or cellular compartments. Members of the Prx1 and Prx6 subfamilies form B-type dimers, with most oligomerizing to decamers via the A-type interface. Members of the BCP-PrxQ, Prx5, TPx and AhpE subfamilies can form A-type dimers. Most proteins of the BCP-PrxQ subfamily members are monomers, with a few of them being A-type dimers. The AhpC/Prx1 can reversibly assemble to constitute doughnut-shaped decamers or even higher-order oligomeric structures. Structural studies of the proteins DmPrx-4783 in *Drosophila* and AsPrx-4783 in *Anopheles stephensi* have confirmed their dimeric state [28].

## 4. Variation of Prxs Among Different Insect Species

A comprehensive literature search revealed that Prxs have been identified in various insect species that belong to the orders Diptera, Lepidoptera, Coleoptera, Hemiptera, Orthoptera and Hymenoptera. The number of Prx genes that have been identified varies across insect species (Table 1). The most extensively studied Prxs are found in the Diptera, *Drosophila melanogaster*. All six Prx families found in mammals have also been identified in *D. melanogaster* (Drosophilidae) [29,30,31]. Prx genes have been identified in dipteran insects such as *D. melanogaster*, *A. stephensi* and *G. morsitans morsitans*. The identified 2-Cys Prx genes include *DPx-4156*, *DPx-4783* and *DPx-5037* from *D. melanogaster*; *Gmm-0929*, *Gmm-3099*, *Gmm-2058* and *Gmm-0601* from *G. morsitans morsitans*; and *AsPrx-4783* from *A. stephensi*. *DPx-2540* and *DPx-6005* from *D. melanogaster*, and *Gmm-2087* and *Gmm-2619* from *G. morsitans morsitans* belong to 1-Cys Prx genes [28,30,32]. *Gmm-2087*, *Gmm-2619*, *Gmm-3099*, *Gmm-2058* and *Gmm-0601* identified in *G. morsitans morsitans* correspond to *DPx-6005*, *DPx-2540*, *DPx-4783*, *DPx-4156* and *DPx-5037* found in *D. melanogaster* [32]. *AsPrx-4783*, the sole Prx gene identified in *A. stephensi*, is an ortholog of *DPrx-4783* from *D. melanogaster*. *DPrx-4783* is believed to protect against oxidative damage caused by intracellularly generated ROS during metabolism [30]. However, *AsPrx-4783* exhibits differences in host cell protection compared to *DPx-4783*. *AsPrx-4783* has been shown to protect *A. stephensi* cells against stresses relevant to malaria parasite infection in vivo, such as nitric oxide (NO), H_2_O_2_, nitroxyl and peroxynitrite [28].

In Lepidoptera, Prx genes have been identified in *Bombyx mori*, *Chilo suppressalis*, *Plodia interpunctella*, *Grapholita molesta*, *Antherea pernyi*, *Spodoptera litura* and *Helicoverpa armigera*. Lepidopteran species show particularly variable reported Prx numbers. For example, only one Prx gene has been identified in *H. armigera* [33], *G. molesta* [34] and *P. interpunctella* [35], while two Prx genes have been identified in *A. pernyi* [36,37] and *S. litura* [38,39], and three genes have been identified in *C. suppressalis* [40,41], though these likely represent minimum estimates given technical limitations in genome annotation and condition-specific expression patterns. In Hymenoptera, four Prx genes have been identified in *A. cenara cenara* [42,43,44,45], while only two Prx genes have been discovered in *Bombus ignites* [46]. In Orthoptera, one Prx gene has been identified in *Gryllotalpa orientalis* [47]. It remains unclear whether the variation in the number of Prx genes among different orders reflects the true diversity of gene numbers or if it is due to the limited study of Prx genes in these species, with some genes possibly yet to be discovered.

## 5. Expression and Distribution of Prxs in Insects

### 5.1. Localization and Tissue-Specific Expression of Prxs

Among the Prx genes identified in various insects, many of them have been studied for their expression patterns in different tissues (Table 1). The most extensively studied among the identified Prx genes in terms of expression patterns are those from *D. melanogaster* (referred to as TPxs in this insect) [30]. Three distinct types of TPxs have been identified in *D. melanogaster*, namely cytoplasmic TPx (DPx-4783, DPx-2540 and DPx-6005), secretory TPx (DPx-4156) and mitochondrial TPx (DPx-5037), which have unique subcellular localization [30]. These findings suggest that this gene family has diversified to perform different physiological functions. Cellular metabolism provides various sources of H_2_O_2_ in different organelles and compartments. The subcellular localization of Prxs in the lepidopteran *B. mori* reveals specialized functional adaptations to different cellular compartments. Among the five isoforms (Prx 3–6 and TPx1) from *B. mori*, BmPrx4 can secret from the cell and BmPrx3 is primarily located in mitochondria [4], which are the primary intracellular source of ROS. As the powerhouses of the cell, mitochondria perform essential functions in cellular metabolism, including serving as centers for energy production, production of biosynthetic precursors and metabolic waste management [48]. These functions may be related to the role of BmPrx3. Additionally, BmTPx1 and BmPrx6 are located in the cytosol, where they likely neutralize cytoplasmic ROS generated during normal metabolism. Notably, BmPrx5 shows dual localization to both mitochondria and peroxisomes [4], indicating specialized protective functions in these highly oxidative organelles, while its additional cytosolic presence may facilitate redox signaling between compartments.

Prx genes are widely expressed in various insect tissues. Expression data for Prxs from insects are summarized in Table 1. Although *ApPrx-2* is detected in all tissues of *Antherea pernyi*, its expression levels are highest in hemocytes and fat body [36], both of which are usually associated with insect defense responses [49,50]. This suggests that Prxs may play a role in immune responses in insects. Similarly, *ApPrx-1* is ubiquitously distributed across different tissues but shows higher expression in hemocytes and fat body [37]. Both BiPrx1 and BiTPx1 proteins are found in the fat body, midgut, muscle and epidermis of *Bombus ignitus* worker bees, but not in hemolymph, indicating that these proteins are not found in the extracellular space and are therefore not secreted [46]. Initially, BmTPx was found to be absent from the hemolymph of *B. mori* larvae [5]. However, subsequent research revealed the presence of BmPrx4 in the hemolymph as a secreted protein [51].

Tissue-specific expression of Prx genes may be related to their functions. Studies on the response of *C. suppressalis* Prx genes to environmental stresses have shown that *CsPrx5* and *CsPrx6* are expressed across all larval tissues, with the lowest levels in the head. *CsPrx5* exhibits high expression in the epidermis and fat body, while *CsPrx6* shows high expression in the epidermis, fat body and midgut. These variations in expression levels may correspond to their specific physiological functions [41]. The transcriptional level of *TPx* gene in the epidermis is relatively lower than in the midgut of *B. mori* and *G. orientalis* [5,47]. *AccTPx1* exhibits higher transcript levels in the thorax than in the abdomen in adult worker honeybees. This difference may reflect the division of labor in this social insect [45].

**Table 1:** Study on peroxiredoxins in different insects.

Classification	Insect Species	Prx Types	Number of Amino Acids	Spatial Expression Profile/Putative Subcellular Localization	Reference
Diptera: Culicidae	*Anopheles stephensi*	Prx-4783 (2-Cys Prx)	196	Midgut.	[28]
Diptera: Drosophilidae	*Drosophila melanogaster*	Prx5 (2-Cys Prx)	157	Not determined.	[29]
		DPx-4156 (2-Cys Prx)	242	Secretion from the cell.	[30]
		DPx-4783 (2-Cys Prx)	194	Cytosol.	
		DPx-5037 (2-Cys Prx)	234	Mitochondria.	
		DPx-2540 (1-Cys Prx)	220	Cytosol.	
		DPx-6005 (1-Cys Prx)	222	Cytosol.	
		Jafrac1 (2-Cys Prx)	194	11E in the X chromosome.	[31]
		Jafrac2 (2-Cys Prx)	242	62F in the 3L chromosome.	
Diptera: Glossinidae	*Glossina morsitans morsitans*	Gmm-2087 (1-Cys Prx)	222	Flight muscle, fat body and midgut.	[32]
		Gmm-0929 (2-Cys Prx)	168		
		Gmm-2619 (1-Cys Prx)	220		
		Gmm-3099 (2-Cys Prx)	194		
		Gmm-2058 (2-Cys Prx)	246		
		Gmm-0601 (2-Cys Prx)	236		
Lepidoptera: Bombycidae	*Bombyx mori*	TPx1 (2-Cys Prx)	195	Cytosol.	[4]
		Prx3 (2-Cys Prx)	227	Mitochondria.	
		Prx4 (2-Cys Prx)	247	Secretion from the cell.	
		Prx5 (2-Cys Prx)	188	Cytosol, mitochondria and peroxisomes.	
		Prx6 (1-Cys Prx)	223	Cytosol.	
		TPx (2-Cys Prx)	195	Fat body and midgut.	[5]
		Prx4 (2-Cys Prx)	247	Malpighian tubules, integument, ovaries, hemocytes, head, fat body, midgut, testis, silk glands and hemolymph.	[51]
		Prx (1-Cys Prx)	223	Gut, hemocytes, Malpighian tubes, ovaries, silk glands and fat body.	[52]
		Prx5 (1-Cys Prx)	188	Hemocytes, fat body and midgut.	[53]
		Prx3 (2-Cys Prx)	227	Midgut, fat body, silk glands, skin, trachea, head and hemocytes.	[54]
Lepidoptera: Noctuidae	*Helicoverpa armigera*	TPx (2-Cys Prx)	195	Head, epidermis, fat body, hemolymph, midgut, Malpighian tubules, salivary glands and central nervous system.	[33]
	*Spodoptera litura*	TPx (2-Cys Prx)	195	Hemocytes, head and cuticles.	[38]
		Prx5 (2-Cys Prx)	159	Epidermis, fat body and midgut.	[39]
Lepidoptera: Pyralidae	*Plodia interpunctella*	TPx (2-Cys Prx)	175	Not determined.	[35]
Lepidoptera: Saturniidae	*Antherea pernyi*	Prx2 (2-Cys Prx)	228	Hemocytes, fat body, midgut, integument, Malpighian tubules and silk glands.	[36]
		Prx1 (2-Cys Prx)	195	Hemocytes, fat body, midgut, integument, Malpighian tubules and silk glands.	[37]
Lepidoptera: Crambidae	*Chilo suppressalis*	Tpx3 (2-Cys Prx)	227	Integument, midgut, Malpighian tubes and fat body.	[40]
		Prx5 (2-Cys Prx)	189	Fat body, head, epidermis and midgut.	[41]
		Prx6 (1-Cys Prx)	223		
Lepidoptera: Tortricidae	*Grapholita molesta*	TPx (2-Cys Prx)	195	Head, epidermis, midgut, Malpighian tubules, fat body and salivary glands.	[55]
Hymenoptera: Apidae	*Apis cerana cerana*	TPx4 (1-Cys Prx)	219	Head, thorax, abdomen, epidermis, muscle and midgut.	[42]
		TPx5 (1-Cys TPx)	220	Not determined.	[43]
		TPx3 (2-Cys Prx)	242	Brain, epidermis, muscle and midgut.	[44]
		TPx1 (2-Cys Prx)	195	Head, thorax and abdomen.	[45]
	*Bombus ignitus*	TPx1 (2-Cys Prx)	195	Fat body, midgut, muscle and epidermis.	[46]
		Prx1 (1-Cys Prx)	220		
Orthoptera: Gryllotalpidae	*Gryllotalpa orientalis*	GoPrx (1-Cys Prx)	220	Fat body, midgut and epidermis.	[47]
Hemiptera: Aphididae	*Acyrthosiphon pisum*	Prx1 (2-Cys Prx)	193	Not determined.	[56]
Hemiptera: Delphacidae	*Nilaparvata lugens*	Prx (2-Cys Prx)	251	Not determined.	[57]
Coleoptera: Lampyridae	*Pyrocoelia rufa*	Prx (2-Cys Prx)	185	Fat body.	[58]

### 5.2. Developmental Regulation of Prxs

The expression of Prxs varies across different developmental stages of insects. In the dipteran *D. melanogaster*, five *TPx* genes exhibit divergent patterns of transcript accumulation, suggesting different roles during tissue proliferation and differentiation [30]. *DPx5037* and *DPx-6005* exhibit similar patterns, with the highest levels being in embryos and adults, and slightly lower levels at larval and pupal stages. *DPx-2540* and *DPx-4783* mRNA levels exhibit an increase during embryogenesis, followed by a significant decrease, particularly in the case of *DPx-2540*. For the gene *DPx-4156*, its mRNA levels decline in larvae, subsequently increasing in pupal and adult stages [30]. In the lepidopteran *H. armigera*, transcripts of *HaTPx* gene are most abundant in the fifth-instar larvae [33]. In contrast, in the Indian meal moth *P. interpunctella*, the *TPx* gene levels are high during the egg stage but low in the early larval stages [59]. In the case of *C. suppressalis*, *CsPrx5* and *CsPrx6* show their highest expression levels in the egg stage [41]. *CsTPx3* and *CsPrx5* are especially higher expressed during larval stages than adult stages; however, *CsPrx6* mRNA levels are generally higher in the female adults than in the larvae [40,41]. In the Hymenopteran *A. cerana cerana*, *AccTPx1* shows highest expression levels in the 15-day post-emergence adults [45], while *AccTPx3* expression is highest in fourth-instar larvae [44]. The mRNA expression of *AccTPx5* was highest in the first-instar larvae. What is more, *AccTPx5* mRNA is plentiful in the newly emerged adult but then decreases in 7- to 10-day post-emergence adults, suggesting that *AccTPx5* may play an essential role in the early developmental stages of the honeybee [43].

### 5.3. Sex-Specific Expression of Prxs

In some insects and other animals, there are sex differences in the expression levels of *Prx* genes. TPx protein expression levels in the blood fluke *Schistosoma mansoni* are significantly higher in males than in females [60]. In contrast, in the Asian rice borer *C. suppressalis*, the *CsPrx6* gene is expressed at a significantly higher level in female adults than in male adults, whereas *CsPrx5* is expressed roughly equally in both female adults and male adults [41]. In the filarial parasite *Brugia malayi*, the BmTPx2 protein is primarily localized in the ovaries of female adults [61], whereas in the oriental fruit moth *G. molesta*, GmPrx1 is most abundant in the accessory glands of sexually mature males [34]. Additionally, Prxs have been identified as sperm proteins in several insect species, including *Aedes aegypti* [62], *Callosobruchus maculatus* [63], *Cimex lectularius* [64] and *Apis mellifera* [65].

## 6. Physiological Functions of Insect Prxs

### 6.1. Roles in Antioxidation and Electron Donor Requirement

The role of Prxs in antioxidation has been confirmed in various insect species, with particularly extensive research conducted in *D. melanogaster* [30,66]. The antioxidant activity of Prxs is primarily reflected in their ability to remove H_2_O_2_, aliphatic hydroperoxides, aromatic hydroperoxides and peroxynitrite [67,68,69]. As the abundance of Prx proteins increases, the rate of H_2_O_2_ removal also accelerates [37]. When external ROS concentrations become excessively high, the expression of *Prx* genes is upregulated, which is also associated with an increased percentage of the cysteine thiol groups on Prxs being oxidized to cysteine sulfenic acid. This process neutralizes excessive ROS, thereby protecting cells from damage caused by oxidative stress. As cysteine-dependent peroxidases, Prxs do not require additional cofactors. They operate through a unique redox mechanism, utilizing Trx or glutaredoxin-glutathione as direct electron donors to reduce H_2_O_2_, organic hydroperoxides and peroxynitrite [70,71].

The removal of ROS by Prxs depends on hydroperoxide substrates, electron donors and different subtypes of Prxs [72]. The purified recombinant 2-Cys Prxs (DPX-4156, DPX-4783 and DPX-5037) and 1-Cys Prxs (DPx-2540 and DPx-6005) from *D. melanogaster* can reduce H_2_O_2_ in the presence of dithiothreitol (DTT). Especially, the three 2-Cys Prxs are active in the thioredoxin system [30]. The purified recombinant BmTPx from *B. mori* also reduce H_2_O_2_ in the presence of electrons donated by DTT and are shown to be active in the presence of Trx as electron donor [5]. On the other hand, Prx6 exhibits glutathione (GSH) peroxidase activity, and its catalytic mechanism relies on GSH instead of Trx for physiologic reductant [73]. Specifically, Prx6 interacts with the π isoform of GSH S-transferase (GTSH), resulting in the reduction of oxidized Prx6 by GSH and the regeneration of active enzyme [74,75,76].

### 6.2. Roles in Development and Lifespan

Prxs play a crucial role in growth and development of organisms via participating in signal transduction and regulation of cellular metabolism [6]. Their involvement in signaling is mediated in part through their effect on ROS. Among the Prxs, Prx5 is particularly known for its role in developmental regulation. In *D. melanogaster*, significant variations in the levels of Prx5 protein have been observed during development, with peak abundance observed during embryogenesis, puparium formation and the late pupal stage [20]. This pattern is similar to that observed for other ecdysone-responsive genes [77], indicating that Prx5 may be a target in insect ecdysone signaling during development with a role in modulating hormone effects. The importance of Prx5 for development is further underscored by the embryonic lethal phenotype observed in progeny derived from the *Prx5*^−/−^ null mutant [20]. Additionally, treatment with a Prx1 inhibitor or siRNA resulted in 30% of *H. armigera* larvae exhibiting development retardation and pupal deformity, highlighting the critical role of Prx1 in regulating larval growth [78]. Prxs are associated with diapause induction in insects. Transcriptome analysis shows that *LmPrx6* is significantly higher in diapause females compared to non-diapause phenotype females, suggesting that *LmPrx6* is involved in diapause maternal locusts [79]. Injection of ds*LmPrx6* significantly reduced the diapause rate, demonstrating that *LmPrx6* is closely associated with diapause induction in *L. migratoria* [80] (Table 2).

Certain Prxs also play a crucial role in lifespan of insects [85,86]. There are multiple lines of evidence suggesting that the activation of the JNK/FoxO pathway is a common cellular response to oxidative damage across animal phyla. In *Drosophila*, neuronal overexpression of Jafrac1, a *Drosophila* homologue of human *Prx II* (*hPrxII*), extends lifespan in flies. The JNK/FoxO pathway protects neurons from oxidative stress, and extends the lifespan of the flies by induction of *Jafrac1* [87]. Through the antioxidant function, Prxs increase resistance to oxidative stress, thereby prolonging lifespan of insects. Overexpression of *Prx5* in flies also increases resistance to oxidative stress and extends their lifespan. The absence of Prx5 expression potentiates tissue-specific apoptosis induced by oxidants, thereby verifying that the effects of Prx5 on longevity occur via its antioxidant action [20]. The mitochondrial TPx can restore wild-type lifespan in a *Drosophila* model for Friedreich’s ataxia [88]. Additionally, *AccTPx1* may be related to the lifespan of honeybees [45].

### 6.3. Roles in Environmental Stresses

In addition to oxidative stress, Prx levels typically increase significantly when environmental stresses disrupt ROS homeostasis and cellular functions. The role of Prxs in response to environmental stresses, such as heat shock stress, may be through scavenging excessive ROS, as various stresses lead to elevated ROS levels [12,89]. The regulation of Prxs transcripts varies depending on the type of Prxs, different species and stress intensity [90]. The transcription levels of *AccTPx1*, *AccTPx3* and *AccTPx5* are upregulated under both 4 °C and 42 °C stress conditions [43,44,45], similar to the results observed for *GoPrx* and *BmTPx* at 4 °C and 37 °C, indicating that Prxs play a protective role against oxidative stress caused by temperature fluctuations [5,47]. In contrast, the transcription level of *AccTPx3* is downregulated at 16 °C and 25 °C, suggesting that these temperatures may not stimulate the host to produce sufficient ROS, or *AccTPx3* may also be involved in different signal transduction processes compared to other TPxs [44]. Moreover, depending on the type of environmental stressor, TPxs may have both beneficial and harmful effects on cell viability. For example, overexpression of DPx-4783 or DPx-5037 in *Drosophila* S2 cells confers increased resistance to toxicity induced by H_2_O_2_, paraquat or cadmium. In contrast, TPx overexpressing cells are more susceptible to copper and heat stress when compared with control cells [66]. The expression levels of *CsPrx5* and *CsPrx6* are significantly upregulated under temperature stress below 20 °C and above 30 °C [41], corresponding to the occurrence of oxidative stress, increased respiration, oxygen consumption, and metabolic rate caused by the increase in environmental temperature in ectothermic animals [91].

### 6.4. Roles in Cell Apoptosis

Apoptosis is a key mechanism in the dynamic remodeling of body structure during metamorphosis. Apoptosis mainly occurs during the pupal stages in insects, during which the restructuring produces the adult body. Mitochondrial-derived ROS activate apoptosis-related pathways [92], and Prxs have been found to play a role in apoptosis [93]. In the silkworm, an increased expression level of *BmPrx4* has been observed during the pupal stage, consistent with the apoptosis process and the apparent melting of the body [51]. This suggests that *BmPrx4* may be involved in the elimination of ROS during metamorphosis. In response to different stressors, Prxs can either promote cell protection or cell death [66]. In the fruit fly, *TPxs* play a crucial role in preventing apoptosis [20,66]. Acute oxidative stress induced by paraquat increases the incidence of apoptosis in muscle and the digestive tract, and this pattern is quite similar to that observed in aged flies [20,94]. DNA fragmentation is also detected in *D. melanogaster* muscles and the digestive tract under comparable conditions, but in addition a strong DNA fragmentation signal in oenocytes is identified, suggesting that the absence of Prx5 expression potentiates tissue-specific apoptosis induced by oxidants [20]. Similarly, overexpression of *LdmPrx* protects *Leishmania donovani* from H_2_O_2_-induced programmed cell death [95]. These findings suggest that Prxs may function in hormonal pathways, regulating ROS levels and thereby protecting against apoptosis.

### 6.5. Roles in Immune Response

Prxs are both inducible and play a role in the defense system via regulating ROS levels in insects. *D. melanogaster* Prx5 is an immune-related antioxidant enzyme that helps maintain intestinal redox homeostasis, and the expression level of Prx5 is upregulated by FoxO, thereby protecting the intestine from infection [96]. Similarly, the expression of *HaPrx1* is significantly upregulated by FoxO in *H. armigera*, which regulates immune responses in larvae infected with the *H. armigera* single nucleopolyhedrovirus (HearNPV) [78]. Baculoviruses have evolved to infect the host mainly through cells in the midgut; thus, the expression of *Prx1* gene in the midgut may be a response to the accumulation of ROS induced by HearNPV, which may be consistent with the results obtained in other insects, including *B. mori* [33,97] and *D. melanogaster* [92]. *Candidatus* Liberibacter asiaticus (CLas), which is vectored by *Diaphorina citri*, is one of the causative agents of greening disease in citrus, an uncurable, devastating disease of citrus worldwide. Analysis of the CLas gene expression in the gut of adult psyllids demonstrates that CLas express Prx, capable of reducing ROS and reactive nitrogen species (RNS) produced in the process of gut infection, suggesting that Prx provides an increased contribution in the infection process [98]. The role of Prxs in prophylactic immunity is associated with the high expression levels of *Prxs* in locusts [99]. *ApPrx1* is significantly upregulated in response to the pathogens, which shows that *ApPrx1* is inducible and plays a role in the defense system, likely via regulating ROS levels in *A. pernyi* [37].

### 6.6. Roles in Insecticide Resistance

Pesticides can induce oxidative responses, including lipid peroxidation in insects [100,101,102,103,104]. Prxs protect cells from oxidative damage by reducing H_2_O_2_, lipid peroxidation, peroxynitrite and thiyl radicals [41]. Changes in *Prx* expression levels have also been linked to pesticide resistance in *B. mori* and *A. cerana cerana* [81]. The transcript expressions of *AccTPx3* and *AccTPx5* are upregulated in *A. cerana cerana* after phoxim treatment [43,44]. Additionally, *SlPrx5* expression is upregulated after the injection of indoxacarb and metaflumizone [39]. BmPrxs in mitochondria can protect cells even more efficiently than cytosolic Prxs after the application of organophosphorus insecticides [4]. Chlorantraniliprole and spinetoram are two commonly used insecticides to control *C. suppressalis*, both of which cause insects to produce high concentrations of ROS [105]. The expression levels of *CsPrx5* and *CsPrx6* increase significantly in larvae treated with insecticides [41]. As the concentration of insecticides increases, the expression levels of *CsPrx5* and *CsPrx6* rise correspondingly, suggesting that they play a role in the response to insecticides [41]. Powell et al. (2011) found 1.9-fold upregulation of a TPx in the Pyridalyl-resistant cell lines compared to protein levels in sensitive Sf21 cell lines [81]. A positive correlation between *NlPrx* T65549 allele frequency and imidacloprid resistance has been observed in *N. lugens* [57]. These observations suggest that the expression levels of *Prxs* in insects correlate with insecticide resistance.

## 7. Summary and Perspective

Prxs play a pivotal role in regulating intracellular H_2_O_2_ concentrations, sensing oxidative stress and facilitating signal transduction. Their diverse functions are associated with differential expression patterns across developmental stages and tissues in insects [30]. The antioxidative activity of insect Prxs is crucial for protecting insects against ROS-mediated toxicity [54]. Prxs remove H_2_O_2_, organic hydroperoxides and peroxynitrite through enzymatic reactions with either Trx or glutaredoxin-glutathione as immediate electron donors [70]. In addition to their enzymatic roles, Prxs also function as molecular chaperones, assisting in cell repair from oxidative damage [106]. Despite the extensive research on Prxs in insects like *D. melanogaster*, *B. mori*, *A. cerana cerana* and *A. stephensi*, their molecular mechanisms and multifaceted functions in Coleoptera insects remain understudied. Further studies are needed to elucidate the molecular events involved in various physiological activities, including ROS removal, DNA protection and immune functions [84]. An interesting phenomenon about Prx genes is that one Prx knockdown results in a compensatory increase in other Prx expression levels, which may facilitate organism survival [107,108]. Exploring the compensatory effects among different Prxs, as well as the potential trade-offs between their antioxidant and immune functions, could provide deeper insights into the evolution of insect defense mechanisms in response to biotic and abiotic stress conditions. Coleopterans consist of the largest order of insect species, yet there have been very few studies on coleopteran Prxs. The diversity and economic importance of coleopterans warrant further investigation into Prxs in this group of insects. Understanding the structural and functional diversity of Prxs, their regulatory mechanisms, and their interactions with other components of the immune system could lead to novel strategies for pest control and disease management in agriculture. This is particularly important for stored-product coleopteran pests, as traditional insecticides have certain applicability and limitations in controlling pests that directly feed on food.

## Figures and Tables

**Table 2:** Research on the physiological functions of Prxs in different insects.

Predictive Function	Species	Prx Types	Reference
Participate in the growth, development, reproduction and other physiological functions of insects	*Locusta migratoria*	Prx6	[80]
	*Helicoverpa armigera*	Prx1	[78]
Involved in insect resistance to insecticides	*Nilaparvata lugens*	Prx	[57]
	*Bombyx mori* and *Apis cerana cerana*	TPx	[81]
Regulate the immune responses of insects	*Antheraea pernyi*	Prx2	[36]
	*Antheraea pernyi*	Prx1	[37]
	*Drosophila*	Prx3 and Prx5	[82]
	*Drosophila*	Prx	[83]
Involved in resisting oxidative stress conditions of insects	*Grapholita molesta*	Prx1	[34]
	*Spodoptera litura*	Tpx	[38]
	*Chilo suppressalis*	Prx3	[40]
	*Chilo suppressalis*	Prx5 and Prx6	[41]
	*Apis cerana cerana*	Tpx4	[42]
	*Bombyx mori*	Prx5	[53]
	*Bombyx mori*	Prx3	[54]
	*Acyrthosiphon pisum*	Prx1	[56]
	*Tribolium castaneum*	TPx	[84]
